# Supplementary material for: Safety, Efficacy, and Immunogenicity of Therapeutic Vaccines for Patients with High-Grade Cervical Intraepithelial Neoplasia (CIN 2/3) Associated with Human Papillomavirus: A Systematic Review
Source: Cancers (Basel). 2024 Feb 5;16(3):672. doi: 10.3390/cancers16030672 (PMC10854525; doi:10.3390/cancers16030672)
Supplement: Supplementary file 1 [file cancers-16-00672-s001.zip › cancers-2495681-supplementary.pdf]

Supplementary Table S1. Search strategy.

MEDLINE/PubMed	#1 (cervical intraepithelial neoplasia [MeSH Terms] OR neoplasia, cervical intraepithelial [Title/Abstract] OR cervical intraepithelial neoplasms [Title/Abstract] OR intraepithelial neoplasm, cervical [Title/Abstract] OR intraepithelial neoplasms, cervical [Title/Abstract] OR neoplasms, cervical intraepithelial [Title/Abstract] OR intraepithelial neoplasia, cervical [Title/Abstract] OR cervical intraepithelial neoplasia, grade III [Title/Abstract] OR cervical intraepithelial neoplasia grade II [Title/Abstract] OR high grade cervical intraepithelial neoplasia [Title/Abstract] OR CIN [Title/Abstract])<br>#2 (papillomaviridae [MeSH Terms] OR human papillomavirus [Title/Abstract] OR human papilloma viruses [Title/Abstract] OR papilloma virus, human [Title/Abstract] OR papilloma viruses, human [Title/Abstract] OR virus, human papilloma [Title/Abstract] OR viruses, human papilloma [Title/Abstract] OR HPV [Title/Abstract] OR HPV, human papillomavirus viruses [Title/Abstract] OR human papillomavirus viruses [Title/Abstract] OR papillomavirus viruses, human [Title/Abstract] OR virus, human papillomavirus [Title/Abstract] OR viruses, human papillomavirus [Title/Abstract])<br>#3 #1 AND #2<br>#4 (vaccines [MeSH Terms] OR vaccine [Title/Abstract] OR immunomodulation [MeSH Terms] OR immunomodulatory therapy [Title/Abstract] OR therapy, immunomodulatory [Title/Abstract] OR cancer vaccines [MeSH Terms] OR vaccines, neoplasm [Title/Abstract] OR tumor vaccines [Title/Abstract] OR injection, therapeutic vaccine [Title/Abstract] OR vaccinotherapy [Title/Abstract] OR therapeutic vaccine [Title/Abstract] OR vaccine immunogenicity [Title/Abstract] OR antigenicity, vaccine [Title/Abstract] OR vaccine immunogenicity [Title/Abstract])<br>#5 #3 AND #4<br>#6 (randomized controlled trial[pt] OR controlled clinical trial[pt] OR randomized[tiab] OR placebo[tiab] OR clinical trials as topic[mesh:noexp] OR randomly[tiab] OR trial[ti] NOT (animals[mh] NOT humans [mh]))<br>#7 #5 AND #6
Web of Science	#1 ALL=((cervical intraepithelial neoplasia OR neoplasia, cervical intraepithelial OR cervical intraepithelial neoplasms OR intraepithelial neoplasm, cervical OR intraepithelial neoplasms, cervical OR neoplasms, cervical intraepithelial OR intraepithelial neoplasia, cervical OR cervical intraepithelial neoplasia, grade III OR cervical intraepithelial neoplasia grade II OR high grade cervical Intraepithelial neoplasia OR precancerous conditions OR preneoplastic condition OR premalignant OR preneoplastic OR CIN))<br>#2 ALL=((papillomaviridae OR human papillomavirus OR human papilloma viruses OR papilloma virus, human OR papilloma viruses, human OR virus, human papilloma OR viruses, human papilloma OR HPV OR HPV, human papillomavirus viruses OR human papillomavirus viruses OR papillomavirus viruses, human OR virus, human papillomavirus OR viruses, human papillomavirus))<br>#3 ALL=((vaccine OR immunomodulatory therapy OR therapy, immunomodulatory OR vaccines, neoplasm OR Injection, therapeutic vaccine OR vaccinotherapy OR therapeutic vaccine OR vaccine immunogenicity OR antigenicity, vaccine OR vaccine immunogenicity))<br>#4 ALL=((randomized controlled trial OR controlled clinical trial OR controlled clinical trials, randomized OR randomized controlled trials OR random allocation OR clinical trial OR clinical trials))<br>#5 #1 AND #2 AND #3 AND #4

SCOPUS	TITLE-ABS-KEY (cervical intraepithelial neoplasia OR neoplasia, cervical intraepithelial OR cervical intraepithelial neoplasms OR intraepithelial neoplasm, cervical OR intraepithelial neoplasms, cervical OR neoplasms, cervical intraepithelial OR intraepithelial neoplasia, cervical OR cervical intraepithelial neoplasia, grade III OR cervical intraepithelial neoplasia grade II OR high grade cervical Intraepithelial neoplasia OR precancerous conditions OR preneoplastic condition OR premalignant OR preneoplastic OR CIN) AND (TITLE-ABS-KEY (papillomaviridae OR human papillomavirus OR human papilloma viruses OR papilloma virus, human OR papilloma viruses, human OR virus, human papilloma OR viruses, human papilloma OR HPV OR HPV, human papillomavirus viruses OR human papillomavirus viruses OR papillomavirus viruses, human OR virus, human papillomavirus OR viruses, human papillomavirus) AND (TITLE-ABS-KEY (vaccine OR immunomodulatory therapy OR therapy, immunomodulatory OR vaccines, neoplasm OR Injection, therapeutic vaccine OR vaccinotherapy OR therapeutic vaccine OR vaccine immunogenicity OR antigenicity, vaccine OR vaccine immunogenicity) AND (TITLE-ABS-KEY (randomized controlled trial OR controlled clinical trial OR controlled clinical trials, randomized OR randomized controlled trials OR random allocation OR clinical trial OR clinical trials)
LILACS	(tw:(neoplasia intraepitelial cervical OR neoplasia cervical intraepitelial OR neoplasia intraepitelial cervical grau III OR cervical intraepithelial neoplasia OR cervical intraepithelial neoplasia, grade III OR NIC OR CIN OR lesão pré-maligna OR lesão pré-neoplásica OR cervical intraepithelial neoplasia grade II OR high grade cervical intraepithelial neoplasia OR premalingant disorder OR preneoplastic)) AND (tw:(papillomaviridae OR human papilomavírus OR papilomavirus humano OR HPV OR human papillomavirus viruses OR human papillomavirus virus OR papillomaviridae)) AND (tw:(vaccine OR therapeutic vaccine OR adjuvant OR adjuvantes imunológicos OR vacinas anticâncer OR cancer vaccines OR vacinas antitumor OR vacinas antitumorais OR vacinas contra o câncer OR vacinas contra neoplasias OR vacinas contra tumores OR immunomodulatory therapies OR therapies, immunomodulator OR vacina terapêutica OR therapeutic vaccine OR vaccinotherapy)) AND (tw:(randomized controlled trial OR controlled clinical trial OR randomized controlled trials OR estudo randomizado))
CENTRAL	#1 (cervical intraepithelial neoplasia OR neoplasia, cervical intraepithelial OR cervical intraepithelial neoplasms OR intraepithelial neoplasm, cervical OR intraepithelial neoplasms, cervical OR neoplasms, cervical intraepithelial OR intraepithelial neoplasia, cervical OR cervical intraepithelial neoplasia, grade III OR cervical intraepithelial neoplasia grade II OR high grade cervical intraepithelial neoplasia OR precancerous conditions OR preneoplastic condition OR premalignant OR preneoplastic OR CIN)<br>#2 (papillomaviridae OR Human papillomavirus OR human papilloma viruses OR papilloma virus, human OR papilloma viruses, human OR Virus, human papilloma OR viruses, human papilloma OR HPV OR HPV, human papillomavirus viruses OR human papillomavirus viruses OR papillomavirus viruses, human OR virus, human papillomavirus OR viruses, human papillomavirus)<br># 3 (vaccine OR immunomodulatory therapy OR therapy, immunomodulatory OR vaccines, neoplasm OR injection, therapeutic vaccine OR vaccinotherapy OR therapeutic vaccine OR vaccine immunogenicity OR antigenicity, vaccine OR vaccine immunogenicity)<br>#4 (randomized controlled trial OR controlled clinical trial OR controlled clinical trials, randomized OR randomized controlled trials OR random allocation OR clinical trial OR clinical trials OR random OR prospective studies OR case control studies OR control OR prospective study OR prospective)<br>#5 #1 AND #2 AND #3 AND #4
EMBASE	#1 ('uterine cervix carcinoma in situ'/exp OR 'intraepithelial neoplasia'/exp OR 'cervical intraepithelial neoplasia 3'/exp OR 'cervical intraepithelial neoplasia 2'/exp OR 'high grade intraepithelial neoplasia'/exp OR 'precancer'/exp) AND<br>#2 ('wart virus'/exp OR 'papillomaviridae'/exp OR 'papillomavirus infection'/exp OR 'papillomavirus-transformed cell line'/exp OR 'human papillomavirus type 16'/exp OR 'human papillomavirus type 18'/exp OR 'human papillomavirus type 16-transformed cell line'/exp OR 'human papillomavirus type 18-transformed cell line'/exp) AND

	# 3 ('vaccine'/exp OR 'immunomodulatory activity'/exp OR 'adjuvant'/exp OR 'vaccination'/exp OR 'virus vaccine'/exp OR 'wart virus vaccine'/exp) AND<br>#4 ('randomized controlled trial'/exp OR 'randomization'/exp OR 'controlled clinical trial'/exp OR 'clinical trial'/exp)

Supplementary Table S2. Data extraction form.

Study: xx	
ROBINS-I: ____________	Cochrane Risk of Bias (Rob 2) Tool: ___________
Authors	
Title	
Year of publication	
Conflict of interest	
Study sponsorship	
Country	
Hypotheses/Aim	
Methods	**Study design:**<br>**Study location:**<br>**Sample size / Sampling:**<br>**Inclusion criteria:**<br>**Exclusion criteria:**<br>**Ethical aspects:**<br>**Trial Register:**<br>**Trial arms:**—Experimental Group: / —Control Group:<br>**Randomization:**<br>**Masking:**<br>**Intervention protocol:**<br>**Per-protocol and modified intention-to-treat analyses:**<br>Per-protocol:<br>Intention-to-treat:<br>**Procedures for data collection:**<br>**Data collection period:**<br>**Procedures:**<br>**Instruments for data collection:**<br>**Outcomes:**<br>Primary outcome:<br>Secondary outcome:<br>**Data analysis:**<br>Statistical analysis:<br>Analysis of experimental data:

Main Results	*Safety:*<br>*Efficacy results:*<br>*Immunological responses:*
Limitations	
Conclusions	
Implications for clinical practice	

Supplementary Table S3. Characteristics and protocols of therapeutic vaccines used in the studies included in the systematic review.

Citation	Vaccine type	Route of administration	Antigen	Intervention protocol
Sheets et al., 2003 [30]	DNA vaccine<br>(ZYC101)	Subcutaneous or intramuscular	HPV 16 E7	15 patients received the vaccine every 3 weeks at the following doses:<br>50 µg group, n=6<br>100 µg group, n=6<br>200 µg group, n=3
Garcia et al., 2004 [31]	DNA vaccine<br>(ZYC101a)	Intramuscular	HPV 16 e 18—E6 e E7	Week 0:<br>100 µg ZYC101a, n=53<br>200 µg ZYC101a, n=58<br>Placebo (saline solution), n=50<br>Week 3:<br>100 µg ZYC101a, n=53<br>200 µg ZYC101a, n=58<br>Placebo (saline solution), n=50<br>Week 6:<br>100 µg ZYC101a, n=51<br>200 µg ZYC101a, n=54<br>Placebo (saline solution), n=48

Garcia-Hernández et al., 2006 [32]	Recombinant viral vector vaccine (MVA E2)	Direct injection into uterus	Oncoprotein E2	54 patients were distributed into a control group and who received therapeutic vaccine. The protocol lasted six weeks and injections were given once a week<br>Group MVA E2 (10^7 particles viral per dose), n=34<br>Control Group, n=20
Kaufmann et al. 2007 [33]	Recombinant viral vector vaccine (HPV 16 L1E7 CVLP)	Injection—did not specify the via	HPV 16 L1 e E7	36 patients were randomized into three groups and received two doses of the vaccine at different concentrations:<br>High dose group that received 250μg of vaccine, n=12<br>Low dose group of 75 μg of vaccine, n=12<br>Placebo group that received 500 μg of albumin, n=12
Roman et al., 2007 [34]	Recombinant bacterial vector vaccine (SGN-00101)	Subcutaneous	HPV 16 E7	21 patients received 500 μg of the vaccine, 4 times with 3-week intervals between each dose
Trimble et al., 2009 [35]	DNA vaccine (pNGVl4A-Sig/E7(detox)/HSP70	Intramuscular	HPV 16 E7	15 patients received the vaccine on days 0, 28 and 56 at different concentrations:<br>0.5 mg group, n=3<br>1 mg group, n= 3<br>3mg group, n= 3
Brun et al., 2011 [36]	Recombinant viral vector vaccine (TG4001)	Subcutaneous	HPV 16 E6 e E7	Vaccine day 1: 21 patients received 5 x 10^7 pfu of TG4001<br>Vaccine day 8: 21 patients received 5 x 10^7 pfu of TG4001<br>Vaccine day 15: 21 patients received 5 x 10^7 pfu of TG4001
Solares et al., 2011 [37]	Peptide vaccine (CIGB-228)	Subcutaneous	HPV16 E7	Vaccine CIGB-22:<br>7 patients received the vaccine 4 times with an interval of one week between each one

Van Steenwijk et al., 2012 [38]	Peptide vaccine (HPV16-SLP)	Not specified	HPV 16 E6/E7	Vaccine HPV16-SLP: 5 patients received 300 µg of the vaccine twice, with an interval of three weeks between one administration and another<br>Placebo group: 4 patients received phosphate buffered saline (PBS), twice, with an interval of three weeks between one administration and the other
Kawana et al., 2014 [39]	Recombinant bacterial vector vaccine (GLBL101c)	Oral	HPV 16 E7	10 patients received the vaccines at weeks 1, 2, 4 and 8 at the following concentrations:<br>1 capsule a day (250 mg), n=1<br>2 capsules a day, n=3<br>4 capsules a day, n=10 (3 from the first phase + 7 added later)<br>6 capsules a day, n=3
Kim et al., 2014 [40]	DNA vaccine (pGX-188E)	Intramuscular	HPV 16/18—E6/E7	9 patients received 3 vaccines at weeks 0, 4 and 12 at the following doses:<br>Dose 1 mg, n=3<br>Dose 2 mg, n=3<br>Dose 4 mg, n=3
Greenfield et al., 2015 [41]	Peptide vaccine (Pepcan)	Intradermal	HPV16 E6	24 patients received 4 vaccines every three weeks in the following groups:<br>Dose 50 µg, n=6<br>Dose 100 µg, n=6<br>Dose 250 µg, n=6<br>Dose 500 µg, n=6
Trimble et al., 2015 [42]	DNA vaccine (VGX-3100)	Intramuscular by electroporation	HPV 16/18—E6 e E7	Group [VXG-3100: 114 patients received 6 mg of VGX-3100 followed by electroporation<br>Placebo group: 40 patients received sterile water followed by electroporation

Alvarez et al., 2016 [43]	DNA vaccine (pNGVL4a-CRT/E7(detox)	Intradermal, Intramuscular or Injection in the cervical lesion	HPV 16 E7	27 patients received the vaccine at weeks 0, 4 and 8 by 3 different routes<br>Intradermal:<br>8 µg, n= 3<br>16 µg: n=6<br>Intramuscular:<br>1 mg, n=3<br>3 mg, n=6<br>Intralesional:<br>1 mg, n=3<br>3 mg, n=6
Coleman et al., 2016 [44]	Peptide vaccine (Pepcan)	Intradermal	HPV 16 E6	50 µg group: 6 patients received the PepCan vaccine + 10 added at the end of the research<br>100 µg group: 6 patients received the PepCan vaccine at this concentration<br>250 µg group: 6 patients received the PepCan vaccine at this concentration<br>500µg group: 6 patients received the PepCan vaccine at this concentration
Choi et al., 2020 [45]	DNA vaccine (GX-188E)	Intramuscular by electroporation	HPV 16/18—E6 e E7	Group [GX-188E]: 34 patients received 4mg of GX-188E followed by electroporation<br>Control group: 36 patients received 1mg of GX-188E followed by electroporation
